# Supplementary material for: Impression formation stimuli: A corpus of behavior statements rated on morality, competence, informativeness, and believability
Source: PLoS One. 2022 Jun 3;17(6):e0269393. doi: 10.1371/journal.pone.0269393 (PMC9165857; doi:10.1371/journal.pone.0269393)
Supplement: Attachment — Submitted filename: Response to Reviewers.doc [file pone.0269393.s001.doc]

School of Psychological Science

35 Stirling Highway, CRAWLEY

Perth, Western Australia, WA 6009

T	+61 (0)8 6488 2688

F	+61 (0)8 6488 1006

E	nicolas.fay@gmail.com

CRICOS Provider Code: 00126G

17 May 2022

The Academic Editor, *PLOS ONE*

Dear Professor Galak,

Thank you for overseeing the review of our manuscript. We are delighted by the positive assessment it received from the reviewers, and welcome their feedback. The manuscript has been revised in light of the reviewer comments. We are grateful to the reviewers for their valuable feedback.

1. **R1** wondered whom the Corpus was constructed for, and why the research was conducted in the US.

The Corpus is likely to be best suited to English speakers from WEIRD (Western, educated, industrialized, rich, democratic) countries, who (for better or worse) are the focus of most psychology research. The Corpus could be used in studies focusing on other populations, but its suitability would need to be tested in a replication/extension of the present study. A US sample was chosen for convenience, and this has been clarified in the manuscript (line 96, p6).

2. **R1** asked whether there were any socio-demographic questions apart from gender and age, and whether gender and age were controlled for in the analyses.

We recorded additional socio-demographic information from Prolific, namely country of birth and first language, but it was not informative as nearly all the participants were born in the US and had English as a first language. We did not control for gender or age in our analyses, as this was outside of the focus of the study. However, the data is available on the OSF, so interested readers can examine the impact of these factors.

3. **R1** asked why we used three of the five morality dimensions.

The number of morality dimensions varies in previous research, ranging from three to five moral content domains (Rozin et al., 1999; Haidt & Joseph, 2004, Graham et al., 2011). While we selected three of Graham et al.’s (2011) five morality dimensions, it was not our aim to have sharp moral content boundaries, as this was beyond the scope of our study; instead we examined moral extremity and its influence on impression formation.

4. **R1** quoted "we generated five categories of statements" and asked who is we, authors? How did you generate these statements, please explain the process in more detail.

The manuscript has been updated to clarify: “To help ensure sufficient variation across each dimension, the authors brainstormed statements from across five categories:...” (line 110-111, p6).

5. **R1** wondered why the number of morality statements (48x2) and competence statements (20x2) we generated were unequal.

Our primary dimension of interest was morality due to its greater influence on impression formation. This is reflected in the greater number of morality statements compared to competence statements. However, it is worth noting that these categories were only used to help us generate the statements (as noted in the manuscript), and it was not the case that each statement was relevant to a single dimension. For instance, participants’ ratings show that the “morality” statements also varied in competence.

6. **R1** asked why a 9-point Likert scale was used (as opposed to a scale with fewer points).

The use of a 9-point Likert scale allows for higher resolution responses than a standard 5-point scale. Pre-testing indicated that participants had no difficulty using this scale.

7. **R2** suggested the literature review could have highlighted how the four dimensions are similar to other concepts from the trustworthiness/person perception literature.

These dimensions are often synonymous to other concepts from the broader person perception literature (see Abele et al., 2021 for a review). This is not to say that there is not occasional nuance, but this nuance is beyond the scope of the current study.

8. **R2** noted that the behavior statements are quite general, whereas most trustworthiness studies are context-specific (e.g., may only use statements about behaviors that occurred in a workplace).

Prior studies use context-general statements like ours. For instance, a proportion of the behavior statements used were drawn from previous studies (n = 66) with the remaining behavior statements generated by the authors to be consistent with previous studies. Despite this, the behavior statements do generate a wide range of responses in terms of morality, competence, believability, and informativeness, which was the main goal of the current study. In keeping it non-specific, it is the authors’ view that it has broader use.

9. **R2** requested more information on why each participant rated 40 behavior statements and each behavior statement was rated by 100 participants.

Each behavior statement was rated by 100 people (as opposed to fewer) to ensure a precise measure for every behavior statement on each dimension. Participants were asked to rate no more than 40 behavior statements to minimise fatigue (which could lead to unreliable responding). The manuscript has been updated to say ‘Pre-testing indicated that participants could become fatigued if they rated more statements than this.’ (line 124-125, p7).

10. **R2** asked if there was a 0 option in the -4 to 4 Likert scale, and wondered why we used a -4 to 4 scale for morality and competence but a 0 to 8 scale for believability and informativeness.

There was a 0 option in the -4 to 4 scale, and we have now made this explicit in the manuscript (line 134-138, p7). We used the two different scales because this was clearer to participants in pretesting, given that, conceptually, morality and competence ranged from negative to positive while believability and informativeness ranged from zero to a lot. The -4 to 4 Likert scale is also similar to other scales used to determine valence judgements (e.g., see Cone et al., 2019).

11. **R2** requested that we order table 1 by something (e.g., the first column).

This has been done, with the table now ordered by the morality (i.e., first numeric) column. Our hope, if it is possible, is that the interactive version of the table can be incorporated into the online PLOS ONE publication. The code for the interactive table is included in the revision documents.

We thank the reviewers for their time and very valuable feedback on our manuscript - we believe the suggestions have improved the manuscript. We hope these changes to the manuscript are sufficient to warrant publication in *PLOS ONE*.

Best regards

Nicolas Fay
